# A case report of effective treatment of intracranial atherosclerotic stenosis treated with the integration of traditional Chinese medicine and Western medicine

**DOI:** 10.1097/MD.0000000000040055

**Published:** 2024-10-11

**Authors:** Wen-Wen Hu, Yiran Chen, Cheng-Ke Cai, Jian Li, Zheng-Guang Chen, Li-Qun Zhong

**Affiliations:** a Dongzhimen Hospital, Beijing University of Chinese Medicine (BUCM), Beijing, China; b School of Cancer and Pharmaceutical Sciences, Faculty of Life Sciences and Medicine, King’s College London, London, UK; c School of Chinese materia medica, Beijing University of Chinese Medicine (BUCM), Fangshan District, Beijing, China; d School of Chinese Medicine, Beijing University of Chinese Medicine (BUCM), Fangshan District, Beijing, China.

**Keywords:** case report, integration of Chinese and western medicine, intracranial atherosclerotic stenosis (ICAS)

## Abstract

**Rationale:**

Intracranial atherosclerotic stenosis (ICAS) represents a prevalent global cause of stroke, posing a notably higher risk of stroke recurrence than other stroke etiologies. Herein, we report a case of a 39-year-old male patient diagnosed with ICAS, treated through an integrated approach incorporating Chinese and Western medicine with significant efficacy and satisfied clinical safety.

**Patient concerns:**

This patient presented with 1 transient ischemic attack and prolonged headache, dizziness and poor sleep quality. In addition, the patient refused to undergo surgery due to the high cost and postoperative risks.

**Diagnoses:**

Diagnostic methods used to identify ICAS include conventional cerebral angiography, magnetic resonance angiography (MRA), CT angiography (CTA), transcranial Doppler ultrasound (TCD), and High-Resolution Magnetic resonance imaging. Considering the cost and risks associated with conventional angiography, noninvasive imaging has emerged as the method of choice for diagnosing ICAS. After a series of noninvasive examinations (CTA, TCD, and HR-MRI), the patient was diagnosed with ICAS.

**Interventions:**

The western medical regimen includes antiplatelet coagulation, blood pressure control, lipid regulation, plaque stabilization, and lifestyle modifications such as exercise, weight loss, and adherence to low-salt, low-fat diets. Complementing this, traditional Chinese medicine (TCM) treatment was guided by the principle of strengthening the spleen, resolving dampness, nourishing blood and harmonizing ying, resolving blood stasis and clearing collaterals. This involved the administration oral Chinese medicine such as modified Shenling Baizhu powder and modified Si Wu decoction.

**Outcomes:**

The efficacy of the treatment was assessed by evaluating the degree of luminal stenosis and peak systolic blood flow velocity in the M1 segment of the left middle cerebral artery (MCA) before and after the intervention. Encouragingly, posttreatment results demonstrated the disappearance of the plaque in the left MCA-M1 segment, with no significant lumen stenosis observed. Moreover, a notable and smooth reduction in blood flow velocity was achieved in the left MCA, indicating positive outcomes from the integrated traditional Chinese and Western medicine approach.

**Conclusion:**

This case report shows that a combination of traditional Chinese and Western medicine is safe and effective in the treatment of ICAS and is worthy of promotion in the clinic.

## 1. Introduction

Intracranial atherosclerotic stenosis (ICAS), also known as intracranial atherosclerotic disease, is a common etiology of ischemic stroke worldwide, especially in Asia.^[1]^ Among Chinese populations, ICAS-related strokes account for 30% to 50% of all ischemic strokes, ranking as a leading cause of death and disability and significant associated morbidity and mortality.^[2–4]^ In addition, ICAS is linked to an elevated risk of Alzheimer disease,^[5]^ which will undoubtedly have a substantial impact on patients’ quality of life and imposing a considerable economic burden on society.

The primary treatment for intracranial arterial stenosis (ICAS) include endovascular and pharmacological approaches, such as primary angioplasty, balloon-mounted stents, stenting, antithrombotic therapy, and risk factor modification. In recent years, neurosurgical methods such as percutaneous transluminal angioplasty and stenting (PTAS) have been widely applied in the treatment of ICAS, but their treatment efficacy varies significantly across different patient cohorts.^[6–8]^ A study has shown^[9]^ that compared with extracranial arteries, intracranial arteries are more tortuous and interconnected, leading to a complex and variable focal hemodynamic environment. This complexity not only increases the difficulty of the surgery and the risk of perioperative complications, but also in-stent restenosis is a significant cause of stroke recurrence or death. Despite most European and American guidelines recommending pharmacologic therapy as the preferred treatment for ICAS, the stroke recurrence rate in ICAS patients remains very high even with active pharmacologic treatment.^[10]^ Against this backdrop, it becomes imperative to explore more complementary therapies for long-term efficacy and safety in clinical settings.^[11]^ Moreover, addressing ICAS necessitates innovative solutions tailored to individual patient profiles and therapeutic responses.

Traditional Chinese medicine (TCM) offers a new perspective on the treatment of ICAS. In TCM, ICAS is classified as a type of “stroke,” common syndromic elements in stroke disease include “wind,” “fire,” “phlegm,” “stasis,” “qi deficiency,” and “yin deficiency.” The main pathological mechanism involves viscera dysfunction and brain collateral impairment, leading to mental function loss. In the Ming Dynasty, Zhang Jingyue proposed that stroke arises from accumulated internal damage, true yin deficiency, and the collapse of primal qi, introducing the “non-wind” theory. Treatment always focuses on differentiating the conditions of yin and yang, deficiency and excess, and the cold and heat of qi and blood. The primary treatment strategy emphasizes tonifying deficiency, especially nourishing true yin. Since the 15th century, TCM has developed renowned prescriptions for treating stroke, including Zuo Gui Wan, You Gui Wan, Da Bu Yuan Jian, and Bu Yang Huan Wu Tang. Modern TCM has developed clinically suitable formulas like Xinglou Chengqi Tang and proprietary Chinese medicines such as Qingkailing injection and Xingnaojing injection, significantly advancing TCM in the field of stroke.

In this article, we shared our experience in treating plaque with stenosis in the M1 segment of the left middle cerebral artery (MCA), a method that not only exempted the patient from endothelial interventions, but also eased the financial burden. We hope that our report sparks new ideas for the treatment of ICAS.

## 2. Case presentation

On February 18, 2021, a 39-year-old Chinese male reported experiencing a sudden onset of right-sided facial asymmetry and transient weakness on the right side of his limbs 10 days prior. The symptoms subsided after 4 hours. Immediately after the seizure, he visited Beijing Tiantan Hospital, where a head Magnetic resonance imaging (MRI) revealed no abnormalities on DWI. However, small patches of low signal on TIWI, high signal on T2W1, and low signal on FLAIR were observed in the left basal ganglia, along with a small soft spot in the same area.

**Figure 1.**
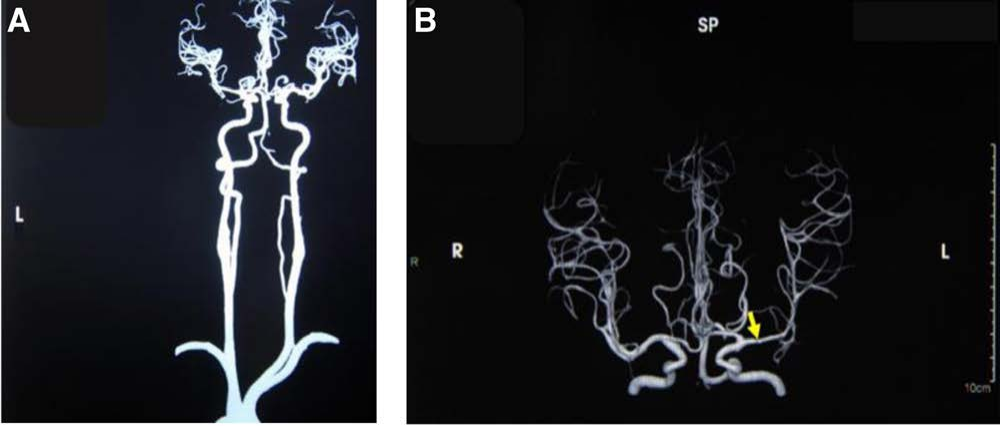
CTA showed that the right vertebral artery was fine throughout (A), and mild-moderate stenosis of the left MCA-M1 segment (B).

**Figure 2.**
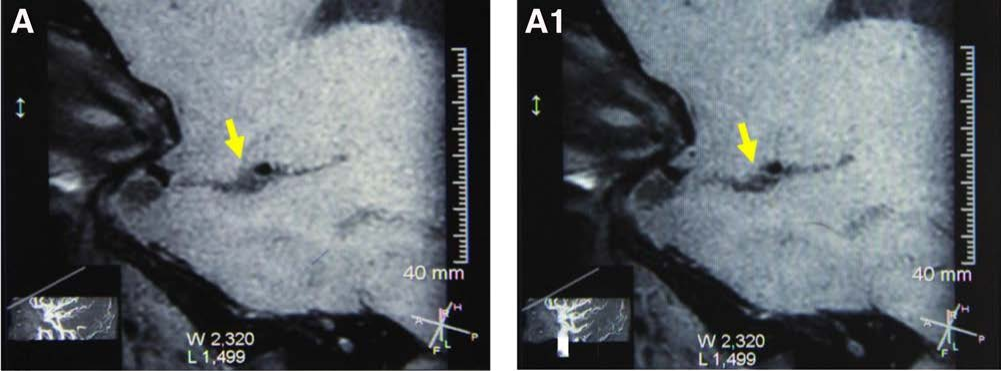
(A and B) HR-MRI showed a circumferential thickening of the wall of the left MCA-M1 segment, and a hypoechoic plaque was visible, accompanied by mild luminal narrowing.

Further inquiry into his medical history revealed that in June 2020, the patient complained of persistent headache and dizziness without obvious triggers. At the time, he sought consultation at Beijing Xuanwu Hospital and underwent a TCD examination. The TCD results indicated moderate stenosis of the left MAC, and mild stenosis of the left terminal segment of internal carotid artery (TICA). Subsequent Cranial CT Angiography (CTA) showed that intracranial atherosclerotic changes, with the left common carotid artery originating from the cephalic trunk, a normal right vertebral artery, and mild-moderate stenosis of the left MCA-M1 segment, as shown in Figure 1A and B. On January 12, 2021, due to the persistent nature of the patient’s headache and dizziness, the patient underwent HR-MRI, which showed that Left MCA-M1 wall plaque with mild lumen narrowing, as presented in Figure 2A and B.

Over the past 6 months, the patient diligently followed a treatment plan involving antihypertensive, antiplatelet, lipid-lowering and plaque stabilization measure. Unfortunately, the therapeutic outcome was unsatisfactory, and he experienced a transient ischemic attack during the course of treatment. While the recent symptoms right-sided facial asymmetry and limb weakness had subsided, he continued to dizziness, headache, and poor sleep quality. Seeking an alternative approach, he turned to Chinese medicine for treatment. The patient reported a 6-month history of hypertension and hyperlipidemia and denied a history of smoking and alcohol consumption.

## 3. Clinical and laboratory examinations

Bilateral tendon reflexes were normal, with pathologic signs (-); the proximal and distal muscle strength of the extremities was grade 5, and the deep and superficial sensation of the distal extremities were symmetrically present, with normal limb muscular tension. There were no problems with eye movements, pupil size or swallowing function.

Serum complement C1q was 141.03 mg/L (normal reference value: 159–233 mg/L), Total cholesterol was 2.8 mmol/L (normal reference value: 3.0–5.7 mmol/L), and low-density lipoprotein cholesterol was 1.26 mmol/L (normal reference value: 2.07–3.1 mmol/L). Triglycerides and high-density lipoproteins were within normal ranges. Various assessment including thrombotic risk assessment, thrombomodulin assay, liver and kidney function, immune function test and rheumatoid factor test yielded normal results.

## 4. Medical intervention

First visit: on February 18, 2021, the patient sought TCM treatment at the Department of Encephalopathy of Dongzhimen Hospital, presenting complaints of dizziness and headache persisting for 6 months, along with a recent transient ischemic attack. He was diagnosed with dizziness – spleen deficiency, dampness, and internalized damp-heat, the treatment approach involved strengthening the spleen, clearing heat and promoting dampness. The patient received modified Shenling Baizhu Powder alongside Western medicine, including oral ramipril (2.5mg/day) for antihypertensive treatment, oral atorvastatin tablets (20mg/day) for lipid regulation and plaque stabilization, and oral aspirin enteric-coated tablets (100mg/day) for antiplatelet coagulation. Regular blood pressure monitoring was recommended to maintain levels below 140/90mm/Hg.

Second visit: after a week of medication, on February 28, 2021, the patient reported continued dizziness and headache that were not obvious in the previous week. The head felt heavy, accompanied by a sensation of tightness. The patient experienced occasional bouts of dizziness and palpitations. During the examination of the tongue and pulse, a red and fat tongue with thin yellow moss was observed. Suspecting insufficiency of vital blood, the presence of phlegm, and blood stasis obstructing the collaterals, the patient was prescribed a modified Si Wu decoction (the main materials include Rhizoma Chuanxiong, Angelicae Sinensis Radix, Radix Rehmanniae and Radix Paeoniae Alba) to nourish blood and Ying, as well as to dissolve blood stasis and clear the collaterals. He followed the same method of administration.

Third visit: On March 14, 2021, the patient experienced significant relief from headache and tightness following the medication. Although occasional dizziness persisted in the morning, fatigue was alleviated by rest. Eating habits were normal, and urination and defecation occurred without issue. The patient reported vivid dreams during sleep. In terms of tongue and pulse examination, there was a red and large tongue with a thin and greasy moss, accompanied by a short pulse and sunken pulse on the left. To enhance the effectiveness of the formula from February 28, we incorporated 10g of Tribulus terrestris to calm the liver and relieve depression, invigorate blood circulation and calm the wind, 12g of Semen Coicis to strengthen the spleen and resolve dampness and 20g of Rhodiola algida to benefit qi and activate blood circulation to further improve dizziness and headache discomfort.

Forth visit: on March 28, 2021, the patient noted a decrease in headache and dizziness, occasional unformed stools, and an absence of sweating. The patient perceived a reduction in fatigue after taking the medication, and experienced fewer dreams than before. The examination of the tongue and pulse revealed a slippery tongue coating and a thin, smooth pulse. To enhance the formula, 20g Amomi Fructus Rotundus and 10g Talcum were added to enhance the efficacy of moving Qi and removing Dampness, and to improve bowel movement.

Fifth visit: during the fifth visit on April 11, 2021, the patient reported a reduction in episodes of headache and dizziness. However, weakness persisted, and there was no sweating. Urination and defecation remained normal. The tongue showed a red tongue with thin moss, and the pulse was fine. The patient was advised to continue with the previous formula. Subsequently, the patient underwent regular follow-ups and received a combination of Chinese medicine to nourish blood and Ying, eliminate blood stasis and clear the channels. This was complemented with Western medicine for blood pressure, reduction antiplatelet therapy, and lipid-lowering to stabilize plaque treatment.

## 5. Outcome and follow-up

Over the span of 2 and a half years of combined Chinese and Western medicine treatment, the patient showed good compliance and tolerance, without encountering any adverse events. Throughout this period, there was no occurrence of a transient ischemic event, and the patient remained free from noticeable symptoms of dizziness, headache and other discomforts. Blood pressure control remained stable, and the patient reported improved sleep compared to before.

**Figure 3.**
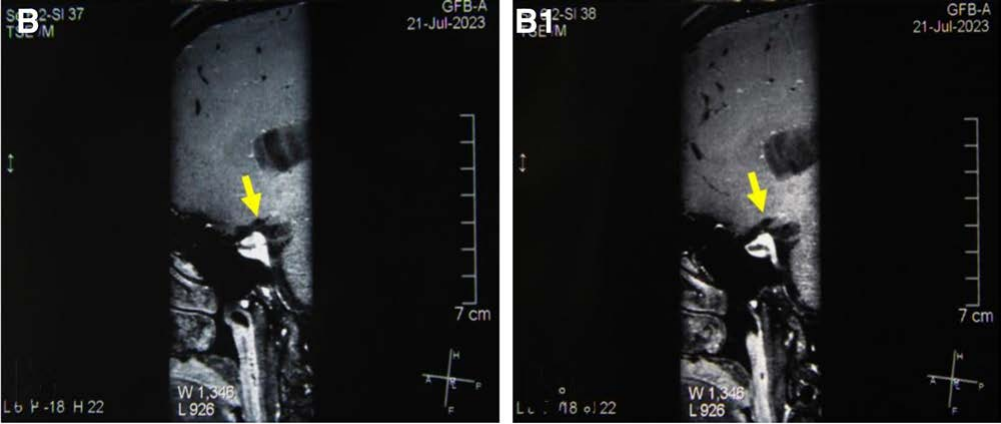
(A and B) HR-MRI was rechecked this year and showed that there was no stenosis in the lumen of the left MCA-M1 segment, and disappearance of the plaque in the M1 segment. (Note: Tiantan Hospital of Beijing changed its examination equipment later, so the magnification was different.)

**Figure 4.**
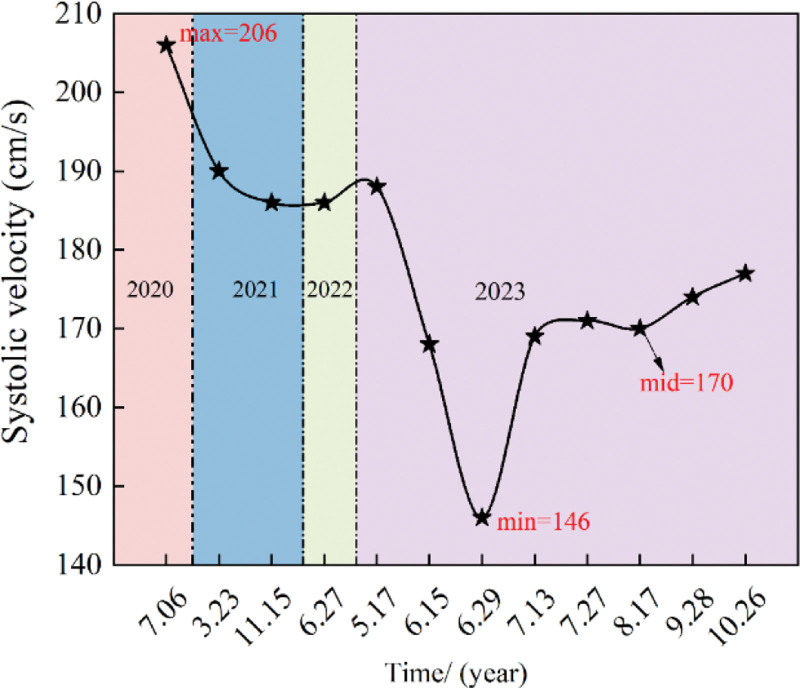
Peak systolic blood flow velocity of the patient’s left MCA was assessed before and during the treatment.

**Figure 5.**
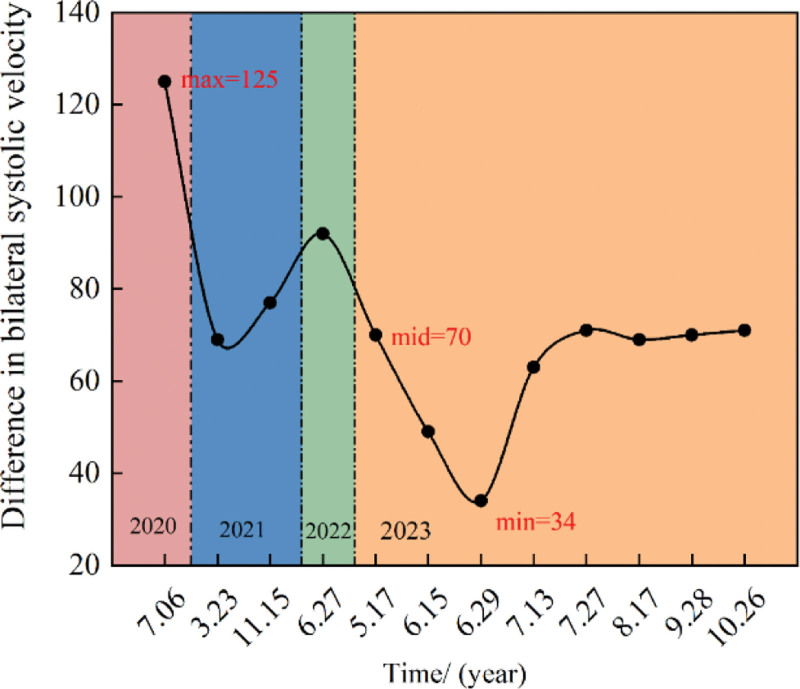
Difference in systolic velocity of the patient’s bilateral MCA was assessed before and during the treatment.

**Figure 6.**
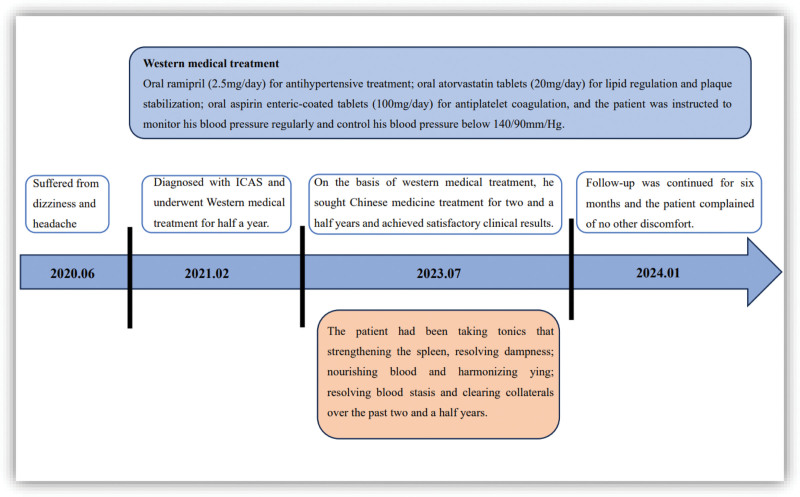
The timeline for medical history.

High-resolution magnetic resonance imaging showed that the lumen of the left MCA-M1 segment did not narrow, and the wall was not significantly thickened after treatment (Fig. 3A and B). TCD results showed a decrease in the peak systolic blood flow velocity of the left MCA transitioned from moderate stenosis to a mild 1. As shown in Figure 4, although the peak systolic blood flow velocity did not reach the normal level, it stabilized at about 170 cm/s. Analyzing the difference in blood flow velocity of the M1 segment of the MCA indicated a maximum difference of 125 initially, gradually decreasing and stabilizing at approximately 70, as shown in Figure 5. These findings suggest that the combined Chinese and Western medicine treatment effectively contributes to increased intracranial blood supply, improved collateral circulation, and the elimination of arterial plaque. The timeline of the medical history is depicted in Figure 6.

## 6. Discussion

In this case of ICAS involving a young male with a chronic course and subacute attack, patients with stroke due to ICAS are usually younger compared to other causes of stroke.^[12]^ Therefore, ICAS should be diagnosed and treated as early as possible by actively controlling vascular risk factors such as hypertension,^[13,14]^ diabetes,^[15,16]^ hypercholesterolemia,^[10,14]^ and smoking.^[12]^

After half a year of treatment with Western internal medicine, the patient did not experience significant improvement and therefore sought help from TCM. During the initial consultation, the patient presented symptoms of dizziness, headache, insomnia, and a history of TIA. Based on the examination of the tongue and pulse, the primary diagnosis was spleen deficiency with excessive dampness and damp-heat transformation. Therefore, the treatment approach included strengthening the spleen and clearing heat and dampness, using a modified Shenling Baizhu Powder. A week after treatment, the patient reported some relief from dizziness and headache, though still experiencing heaviness and tightness in the head. Subsequently, based on the patient’s tongue and pulse indicators, the prescription was adjusted to a modified Si Wu decoction, which alleviated the symptoms. Over 2 and a half years of TCM treatment, the patient continuously took the modified Si Wu decoction. The medication was regularly adjusted according to the specific tongue and pulse readings at different times.

Notably, the basic components of the formula did not change, as shown in Table 1.

**Table 1:** The main TCM prescriptions during the treatment.

Latin name	Chinese name	Individual dosage (g)
Pheretima Aspergillum	Di-Long	20
Corydalis Yanhusuo	Yan Hu Suo	10
Angelicae Sinensis Radix	Dang Gui	10
Rhizome Chuanxiong	Chuan Xiong	10
Aucklandiae Radix	Mu Xiang	10
Radix Rehmanniae	Shu Di	10
Radix Paeoniae Alba	Bai Shao	10
Uncaria Rhynchophylla	Gou Teng	15
Spatholobus Suberectus Dun	Ji Xue Teng	15
Prunella vulgaris Linn	Xia Ku Cao	12
Semen Cassiae	Jue Ming Zi	15
Perilla frutescens	Zi Su Ye	10
Concha Ostreae	Sheng Mu Li	15
Bulbus Fritillariae Thunbergii	Zhe Bei Mu	8
Radix Astragali	Zhi Huang Qi	10
Carapax et Plastruw Testudinis	Gui Jia	15
Glycyrrhizae Radix et Rhizoma	Zhi Gan Cao	10

In this formula, Rhizoma Chuanxiong, Angelicae Sinensis Radix, Radix Rehmanniae and Radix Paeoniae Alba, as constituents of Si Wu decoction, can nourish and invigorate the blood. Di-Long serves as another important medicine to fulfill its role in activating blood circulation, removing blood stasis, and activating channels and collaterals. Currently, Di-Long protein hydrolysis isozymes extracted from Di-long have been recommended as a drug for preventing blood clot formation to address ischemia and hypoxia in vital organs like the heart and brain.^[17]^ Modern pharmacological studies have found that the active ingredients of the Di-Long mainly include amino acids, lipids, nucleotides, organic acids proteins and peptides. Among these lipids, amino acids, and proteins are likely the active substances contributing to Di-Long antithrombotic effect. Di-Long protein is used for the treating ischemic conditions such as stroke,^[18]^ vascular dementia,^[19,20]^ myocardial infarction^[21,22]^ and deep vein thrombosis due to its antithrombotic activity.^[17]^ Uncaria Rhynchophylla and Semen Cassiae calm the Liver and treat dizziness. Spatholobus Suberectus Dun and Aucklandiae Radix enhance the function of nourishing and invigorating blood and opening up collaterals. Prunella vulgaris Linn, Concha Ostreae, Bulbus Fritillariae Thunbergii, and Carapax et Plastruw Testudinis can soften hardness and dissolve phlegm. Perilla frutescens regulates the Qi, broadens the middle Jiao and harmonizes the Stomach. Radix Astragali replenishes Spleen and Lung Qi, while Glycyrrhizae Radix et Rhizoma moderates and harmonizes the other drugs.

We adopted an integrative approach in this case, combining TCM and Western medicine, with TCM playing a key role throughout the treatment process. Compared to conventional Western medical treatments, TCM generally costs less, providing an economical alternative to surgical interventions. TCM is noninvasive, reducing potential complications and the risk of mortality, making it a safer option. Regarding treatment satisfaction, TCM emphasizes individualized treatment and advocates for diagnosis and treatment based on an overall analysis of the patient’s health and lifestyle, which generally enhances patient’s satisfaction and acceptance of the treatment. In terms of long-term efficacy, TCM focuses on the fundamental causes of diseases and long-term conditioning rather than merely symptomatic treatment. This approach helps reduce the recurrence of strokes and can improve patients’ quality of life. From the discussion above, we can see the potential advantages of TCM in treating ICAS. However, there is relatively little scientific evidence supporting the treatment of ICAS with TCM. Future research should validate the effects and mechanisms of TCM treatments and explore better integration with Western medical practices.

## 7. Conclusion

In this report, we present and describe a 39-year-old patient with ICAS, who was treated with a combination of traditional Chinese and Western medicine, resulting in improvement in discomfort, and elimination of plaque in the left MCA-M1 segment. Our report provides some ideas for the treatment of patients with ICAS, especially those in the early stage of ICAS onset.

## Author contributions

**Data curation:** Liqun Zhong.

**Funding acquisition:** Liqun Zhong.

**Investigation:** Wen-Wen Hu.

**Methodology:** Liqun Zhong.

**Project Administration:** Yiran Chen, Cheng-Ke Cai, Jian Li.

**Supervision:** Liqun Zhong.

**Validation:** Cheng-Ke Cai, Zheng-Guang Chen, Liqun Zhong.

**Visualization:** Yiran Chen, Jian Li, Zheng-Guang Chen.

**Writing – original draft:** Wen-Wen Hu.

**Writing – review & editing:** Wen-Wen Hu.
